# Lectures on Displacements of the Uterus

**Published:** 1860-06

**Authors:** E. R. Peaslee

**Affiliations:** Prof. of Obstetrics and Diseases of Women and Children in the New York Medical College


*Lectures on Displacements of the Uterus.* By E. R. Peaslee, M.D., LL.D., Prof. of Obstetrics and Diseases of Women and Children in the New York Medical College.

(Continued from last No. of the Monthly.)

### LECTURE IV.

Gentlemen—To-day I have to speak of the symptoms, the diagnosis, and the treatment of prolapsus uteri.

It is an important practical fact that the first degree of prolapsus is often productive of far more suffering than the third even. Sometimes the malaise is very great during the first degree, but mainly subsides when the second is attained, since the cervix then rests on the perineum, and the strain is taken off the uterine ligaments. Much, however, depends on the social condition of patients affected by this displacement. The lower classes suffer far less from the same degree of descent.

There are few, if any, of the rational symptoms which are distinctive of prolapsus; the physical signs alone being conclusive. And many patients who have not the least degree of prolapsus infer very positively from their sensations that they have this displacement, and their medical adviser may be equally deceived till an internal examination is made. I shall, therefore, pass rapidly over the rational signs of this displacement.

It is, of course, desirable to detect the existence of prolapsus while still in the incipient or first degree. There is reason to believe that this stage often exists a long time, causing great suffering meanwhile; but remains undetected, because the patient does not suspect the cause, and specify the early symptoms. But it is while in this state that prolapsus is most promptly and certainly cured; and when it has become complete, (3d stage) the patient herself has already made out her own case. I shall, therefore, speak more particularly of the rational symptoms of the first and second stages.

1. The most *characteristic* rational symptom of the first stages of prolapsus, is found in the fact that the unpleasant pelvic sensations are usually at once removed, or at least much relieved, when the patient lies down; again to return when she rises, and to be increased by standing or walking.

I shall also specify the most prominent of the rational symptoms produced by this displacement, and refer you for the sympathetic or indirect symptoms, common to this and the other classes of displacements, to the preceding lecture. I should, however, premise that the functions of the uterus are usually not essentially deranged in the first two degrees of prolapsus. Leucorrhœa, perhaps, always exists; but menstruation generally continues regular, and does not become either decidedly painful or menorrhagic. Conception also occurs in these two stages of prolapsus, and sometimes even in the third degree. Churchill quotes a case of even irreducible prolapse in which pregnancy occurred. On the other hand, either degree of prolapse may become the cause of sterility.

Referring to my first lecture for the direct rational symptoms common to the various uterine displacements, I call particular attention here to the following as being most frequently met with in the first degrees of prolapsus. A "bearing-down" sensation, a sense of dragging from the umbilicus, (from tension of the urachus,) or from the loins; a feeling of weight in the anus, increased while standing, and usually ascribed by the patient to piles; pain in the back, extending to the groins; irritation of the bladder and difficult or frequent micturition; and rectal tenesmus.

Very rarely an acute sensibility of the whole surface of the abdomen, simulating peritonitis, occurs in the first stage of prolapsus; and which disappears as soon as the displacement is rectified. Dr. Meigs has seen thirty cases of this kind.

In the third degree of prolapse there is difficulty in micturition and defecation. Sometimes neither of these acts can be effected till the patient lies down, and returns the protruded mass. Retention of the urine, necessitating the use of the catheter, is a common symptom in irreducible cases.

2. A *vaginal* examination should be made in all doubtful cases, while the patient is standing. Cases indeed occur in which the rational symptoms clearly point to prolapsus, but in which not the least descent of the womb can be certainly ascertained, even while the patient is in the erect position. But on raising the womb slightly on the tip of the finger all these symptoms disappear at once, to return again the instant the support is withdrawn.

In the *first* degree of prolapsus—exclusive of the class of cases just alluded to—the vaginal exploration shows the os uteri to be too low in the vagina, and usually in contact with its posterior wall. In the *second* degree the os rests on the perineum, or even presents between the labia; the fundus being now retroverted, as already explained, (Lect. 3d, p. 433.) In both these degrees, pressure applied in the proper direction usually at once restores the uterus to its normal position.

In the *third* degree, or complete prolapsus, the external tumor is somewhat sensitive, firm, and usually fluctuating anteriorly; being six to ten inches long, and sometimes hanging half way down the thighs. It is sometimes of an oval or globular form, but is usually conical, the uterus forming its apex below, while its base fills the os externum above, and the labia extend along its sides. In cases of long standing, however, the lower extremity of the tumor becomes the largest. But even when the extruded mass attains to its largest size, the uterus itself may not be at all enlarged. The os uteri is, of course, *always found at the lower extremity of the tumor*, presenting a rounded or semilunar form, or being sometimes so contracted that a pin will scarcely enter it, (Bochmer.) There is, except during the menstrual period, a constant, and often very abundant, flow from it of mucus, or of pus; and the surface of the tumor, at first presenting the natural color of the mucous membrane, soon becomes dark red, or brown, and is inflamed or ulcerated, or even incrusted with saline deposits. Finally, however, the membrane may become dry, and more and more resemble the skin. The cervix uteri also sometimes becomes very much elongated.

*Diagnosis of Prolapsus.*—There are no rational signs which will certainly distinguish the first two degrees of prolapsus from the other classes of uterine displacements, as we have seen. Usually, however, the feeling of weight in the anus, and of pressure on the bladder, and the strangury, are at once relieved on lying down; which is not the case in anterior and posterior displacements. But the vaginal exploration is conclusive.

Elongation of the cervix has been mistaken for the *first two degrees* of prolapsus; but in that condition the vagina still retains its normal length and firmness. This is the fact also in cases of polypus uteri, partial inversion of that organ, and tumors in the pelvis—all of which have been mistaken for prolapsus. Besides, in these affections the os uteri is not to be felt at the lower extremity of the tumor; and in partial inversion we have the characteristic repeated floodings.

The *third* degree of prolapsus may be mistaken for polypus uteri, complete inversion of that organ, and prolapse of the vagina.

But polypus is not sensitive, has nothing corresponding to the os uteri at its lower extremity, is not reducible, and presents no fluctuation anteriorly, as is the case with reducible prolapse. Moreover, the vagina is not shortened in case of polypus. Sometimes a fissure exists at the lower end of a polypus; but this is easily distinguished from the os uteri by the attempt to pass a probe.

Complete inversion is known by the repeated floodings, the constitutional disturbance, and the absence of the os uteri at the lower extremity of the tumor.

Prolapse of the vagina is a less solid tumor than prolapsus uteri; and here again the os uteri is absent.

*Effects of Prolapsus, and its Terminations.*—If no treatment is resorted to, the first and second degrees of prolapse finally lead to so many sympathetic affections, as explained in the preceding lecture, in addition to the local suffering and the exhausting leucorrhœal discharge, that the health becomes completely deranged, and the patient succumbs; or she may perish in consequence of an inflammation excited by the malposition of the organs within the pelvis.

The third degree of prolapsus may prove fatal from the constitutional irritation consequent on the ulceration of, and the discharge from the extruded mass, or from retention of urine—and especially if it be irreducible.

A fatal termination of prolapsus is, however, quite uncommon. The patient most frequently drags on a miserable existence till carried off by some other disease. Gangrene may occur in complete prolapsus, and the whole uterus slough away, and the patient still recover. And women who suffer constantly from a complete prolapsus, so long as they continue to menstruate, and have, perhaps, been bedridden for years, sometimes experience only an inconvenience from this displacement after menstruation finally ceases, and again find themselves able to stand and walk without much difficulty.

It should also be added that prolapsus of the first two degrees is generally entirely relieved by pregnancy, after the fourth month has passed. The same may also be the case with complete prolapsus, if it remain reducible. And in all cases we may expect that the recurrence of prolapse after parturition may be prevented by judicious management. The occurrence of pregnancy is quite probable so long as even complete prolapse is reducible, and it is possible though the displacement be irreducible; but in the first and second degrees, conception is sometimes prevented by the close contact of the os uteri with the vaginal wall, and hence sterility is an effect of this displacement, as before noted.

If pregnancy continue through the fourth month, it is likely to go on to the full term. This has, indeed, occurred in a case of prolapsus which was irreducible from the period of conception till after parturition had taken place.

*Complications of Prolapsus.*—The prolapsed uterus remaining in a state of hyperæmia, sometimes becomes hypertrophied to twice its normal size; or the cervix may become elongated to twice its normal length. In complete prolapsus, the bladder and rectum may come into contact above the extruded uterus, and adhesions between them, or between the uterus and contiguous parts, may form;* in which case the prolapsus becomes of course irreducible.

Prolapsus may also be complicated with any of its direct causes, (intra- or extra-uterine tumors, inflammation, &c., &c.,) specified in the preceding lecture; and some of these may render it irreducible and incurable. It is also frequently attended by ovaritis; and which requires the treatment appropriate to it in other conditions.

Pregnancy is a not uncommon complication of prolapse, and there is no very great tendency in these cases to miscarriage after the fourth month is passed, as has been already said. The prolapsed uterus can usually be reduced up to the fourth month; and in one instance Giroud effected the reduction but six days before delivery. In some instances, however, the uterus cannot be reduced after the first month of pregnancy.

*Prognosis of Prolapsus.*—We may expect to afford great relief in all cases of prolapsus; and, as a general rule, if we can make the circumstances of the patient accord with our directions, we may expect a complete cure. The prognosis of uncomplicated prolapsus may be more positively expressed in a favorable manner, than that of any other uterine displacement; and, perhaps, no case should, in the most favorable circumstances, be pronounced incurable. The most unpromising and obstinate forms of irreducible prolapsus have yielded to persevering treatment.

In married patients, however, of the lower classes, circumstances very often interfere with an entire recovery, unless pregnancy supervenes.

In case of complications with prolapsus, the prognosis is of course based on the probability of removing such complications, or of neutralizing their agency in producing or perpetuating the uterine displacement.

*Treatment of Prolapsus.*—Prolapsus accidentally discovered, and producing no symptoms—for such cases occur—requires no treatment. But in all cases of moment, local treatment is required; and in those of long standing, general and moral treatment is also necessary.

I. The *local* treatment is of the first importance in all cases, as has already been shown (Lect. 3d, p. 427) the indications being:

1. To remove the cause, if possible, of the displacement;
2. To replace the uterus;

---

*Baillie, Morb. Anat. 4th Ed. p. 419.

3. To maintain it in position by appropriate mechanical appliances, if the natural supports are not sufficient, or until they are sufficient, to maintain it. Cases occur in which the relaxation of the vagina may be overcome in part or entirely by the use of astringent injections by the patient herself, after the uterus is replaced; and in all cases if there be no inflammation or congestion of the latter, the reposition should be at once effected, and then the applications required to retain it *in situ* should be made.

1. The removable causes of prolapsus are (Lect. 3, p. 435,): congestion (or inflammation), hypertrophy, polypus, and arrested involution of the uterus itself; and ascites and ovarian dropsy. And, as a general rule, either of these should be removed before the uterus is replaced. There would also be but very few exceptions to it in the case of accompanying polypus and the two forms of dropsy; but in the case of congestion, inflammation, hypertrophy, or arrested involution, the treatment instituted to remove these conditions will often be more promptly effectual if the uterus is first replaced, and retained *in situ,* so far as a recumbent position will accomplish this; but no *pessary* can be used, as we have seen, while there is congestion or inflammation (Lect. 2, p. 257.)

I shall not here speak of the methods of removing uterine polypi, and dropsy, either ovarian or abdominal, as I have discussed these topics in another connection. The most common complications are, congestion or inflammation of the cervix, or of the entire uterus, and hypertrophy of the cervix. Nor will I here dwell upon the treatment of these conditions. You remember congestion and inflammation are treated mainly by rest in the recumbent position, leeches to, or free scarifications of, the cervix, saline laxatives, vaginal injections of cold flaxseed or slippery elm infusions, several times daily; the hip bath at night, with an appropriate regimen; while hypertrophy is removed by the tinct. Iodine, the potassa cum calce, the acid nitrate of mercury, or the actual cautery.

In chronic cases, however, in which the general health is already failing, it is a matter of the utmost importance to avoid confining the patient to her apartment, or, indeed, within doors, if this can possibly be avoided. In such cases, therefore, you may return the uterus from time to time, while resorting to the above-mentioned means, and keep it in place for a few hours by a medicated pessary, while the patient sits up, or even rides out, if this be expedient. A mass of lint or cotton, of the size, when pretty firmly compressed, of a hen's egg, or larger, completely saturated with a mucilage of gum-Arabic

(or flaxseed infusion,) have been found to answer this purpose well. And while the treatment for the removal of hypertrophy of the cervix is being employed, we may often introduce a pessary and allow the patient to walk about, without retarding the cure.

If ulceration coexist with prolapsus, it may also receive attention after the congestion is removed and the uterus replaced; the application of the nit. Argenti once in six or seven days usually being all the treatment required for its removal. Ulceration will, however, often at once disappear after the malposition is removed, and treatment promises nothing so long as congestion or inflammation persists.

2. Having fulfilled the first indication of the treatment, we may proceed to replace the uterus. In the first and second degrees of prolapsus this is usually at once accomplished by the pressure of the tip of the index finger, carried in the proper direction, while the patient lies upon the back.

In some cases, however, the uterus, falling down about one-half the length of the vagina, becomes fixed to such an extent, that no slight or safe amount of pressure can dislodge it; or, in other words, the prolapse becomes practically irreducible. In such a case, the globe pessary, of a proper size, may be found of great value. If there be space for the introduction of a little more than one-half of the instrument, the constant action of the sphincter vaginæ upon the portion which at first protrudes between the labia, will gradually carry the instrument into the vagina, and the uterus upward. before it, to its normal position. Of course, the patient is to maintain the horizontal position after the instrument is applied, and avoid every effort; and from one to three days may be required to accomplish the complete replacement. Dr. Meigs mentions a case which was relieved in this way within 24 hours, by a globe pessary 1¼ inch in diameter.*

In cases of the *third* degree of prolapsus, the uterus can usually be returned to its normal position in the following manner: the patient lying on the back with the pelvis elevated, apply pressure with the thumb and finger (previously well oiled) to the lowest and central part of the mass, (the cervix uteri,) and endeavor to carry this portion upward in the direction of the axis of the whole mass. The patient must, of course, avoid making any effort during the reposition, and after it is effected, and must still retain the horizontal position. If the tumor is swollen and tender, leeching, and perhaps bloodletting

* Woman and her Diseases, p. 203.

also, or deep incisions, may be necessitated before reduction is attempted. Sometimes the continual pressure of a bandage has been used with final success. Reposition has been in some cases effected by judicious management, after the mass has remained in an irreducible state for years; and we should never despair, in an uncomplicated case, of final success. If there be calculi in the bladder, they should be removed before the reduction is attempted.

Sometimes great distress is produced by the reposition of the uterus. If this continue, under the influence of appropriate anodynes, the mass should be allowed again to extrude and the reduction be deferred to another time. Adhesions existing, may, on reduction of the mass, expose the patient to all the dangers of strangulated hernia.

3. We now come to the third indication of the local treatment, viz., the application of a proper mechanical support to retain the uterus in place. We often meet with cases in which the uterus remains in place after the removal of the cause of the descent, as explained under the first indication, without the aid of any mechanical support. Or an astringent medicated pessary worn for a few days, may be all that is required—as a mass of lint or cotton wet in a solution of tannin (ʒj to aquæ f℥vi.) In cases where there is merely relaxation of the vagina, without any tenderness or congestion of either that canal or the uterus, I have often effected a cure in a few weeks by the application once in 4 to 6 days, through the speculum, of one-half teaspoonful of tannic acid, and then a mass of dry cotton large enough to somewhat distend the vagina, and which remains there till removed for the next application. The cold hip bath at night is also useful in these cases. Meantime the patient walks or rides out daily.

But in most cases of long-standing prolapsus, a mechanical support is constantly required for several weeks or months; and hence a pessary becomes the appropriate instrument. I have specified the conditions in which pessaries may be applied, and have given the rules for their management in the second lecture (p. 257); and will not here repeat them. I have also there given my reasons for preferring the steel spring covered with gutta percha, or the spring of pure tin, in almost all cases of prolapsus.

The introduction of the pessary is effected with the greatest facility while the patient lies on the back; and the steel spring covered with gutta percha is introduced with the least pain, and usually produces the least subsequent irritation.

I shall not, therefore, be understood by any means to recommend a pessary in every case of prolapsus. I agree with Dr. West* that a pessary may be neither necessary nor suitable—

1. In slight cases of prolapsus.

2. When the descent, still comparatively recent, is due to the persistence of the puerperal state of hypertrophy (or arrested involution after parturition.)

3. When uterine disease of whatever kind was the cause of the displacement, and still demands treatment.

4. I also add that we must hesitate to use a pessary in case of a pregnant patient, lest it induce a miscarriage.

If for any reason a pessary cannot be borne, and the patient must still be standing and walking daily, the pressure of a pad upon the perineum by means of an appropriate bandage, as recommended by Dr. Hamilton, may secure great relief as a palliative measure for the time being.

After the pessary is applied, the patient should use an astringent vaginal injection at night (alum ʒj to ʒiv, or tannic acid ʒj to ʒij, to water Oj;) and an injection of water at the temperature of her apartment, in the morning. Some of the other astringents mentioned in the second lecture may be substituted for these; but if there be heat or tenderness of the vagina or of the cervix uteri, the acetate of lead, (ʒj to ʒij, to water, or mucilage Oj,) is preferable, I think, to all the rest.

The pessary should be removed once in two weeks or less, in order to examine and cleanse it, and whenever a smaller one will answer instead, it is to be substituted. In cases of complete prolapsus, an instrument three inches in diameter, and very strong, is usually at first required.

On removing a pessary, the patient should be made aware that she will, for a time, feel a need of its support; this sensation being generally complained of on its removal, whether the instrument is longer required or not.

How long should a pessary be worn, is a question not easily answered in definite terms. Several months at least, in chronic cases, and especially if the patient is past the meridian of life, and has had several children. We can know when it can be dispensed with, only on ascertaining the effects of its removal for a day or two. Pessaries have been found beneficial, and indispensable, for years in succession; but we may hope to diminish such instances to a very small number by judicious treatment, except when some incurable complication coexists.

---

* Lectures on Diseases of Women.

On discontinuing the use of the ring pessary, a small astringent medicated pessary, as before described, or a little muslin bag (a sachet,) filled with tannic acid, or powdered nutgalls, may be used for a time instead. These last can be introduced at night, and also removed in the morning, by the patient herself; if a thread be attached to the sachet previously to its introduction. Astringent vaginal suppositories also, are a good substitute for the sachet just described, for which, the following formula answers well—or the butter of cocoa may be combined with the astringent substance:

℞.—Tannic acid, ℈iiss., or alum, ʒij, or acetat. lead, ʒss.

Ceræ albæ, ʒij.

Axungiæ, ʒvi.

Mix—make iv. suppositories. One to be applied every second or third night.

Such is the local treatment of the three degrees of prolapsus uteri. Certain modifications are, however, required by certain complications. If, in the cases of complete prolapsus, the uterus becomes too heavy from a tumor in its own substance, or is so pressed downward by an extra-uterine tumor, that the pessary I have recommended is not sufficient to sustain it in place, a stem pessary must be used instead. It is in this class of cases that the peculiar value of this form of pessary becomes apparent; and to this class I would restrict its use. In cases of the first and second degrees of prolapsus, complicated with rupture of the perineum, the globe pessary is usually found to be preferable to any other form.

Finally, several operative methods have been proposed to effect the radical cure of reducible prolapsus, which has resisted treatment long applied. I here merely allude to the operation for contracting, and that for entirely closing the vagina, and refer you to the proper authorities for the manner in which they are performed.*

II. The *general* treatment of prolapsus uteri will, of course, depend on the precise condition of each patient. If all the functions are still in a normal state, as is often the fact in recent cases, no medication at all is required.

Generally, however, the digestion is more or less deranged before the patient applies for aid. Constipation exists in almost all cases; and saline laxatives, if there be any heat or tenderness of the parts, or rhubarb and soda, or some other mild aperient, if there is no such symptom, may be required. This symptom removed, the bitter tonics are

---

* See Churchill on Diseases of Women, p. 362, and 366–8.

generally indicated; and iron also, if there be anæmia. You have observed how frequently I have felt it necessary to prescribe the various preparations of iron in this class of cases. Bland, but the most nutritious diet, is also required; and much riding also, with but very little walking. Regular sleep must be secured by the use of valerian or lactucarium at night; or of some other soporific of the same class with them, rather than of opiates. But I can only indicate the general principles under this head, since the peculiarities of each case will suggest its general management.

III. I must add a word in regard to the *moral* treatment of this affection. Your sympathy for the patient, as indicated by your kindness and delicacy of manner, will often accomplish far more than your general treatment; and the hope you inspire by your candid expression of your expectation to relieve, or to cure, is the most efficient of all tonics. Such patients are usually despondent, and the physician should, if possible, keep them cheerful by suggesting appropriate occupation and amusements; and while he sets an example of kindness and sympathy, he should inculcate the same upon the friends of the patient with whom she is in constant relation. Especially should the husband, if the patient has a husband, be made to understand the nature of the case, and why his wife is changed in spirits, and perhaps in temper also, as she is. A husband's sympathy is often, in such cases, worth more to the patient than all the medication; and the latter is often rendered entirely useless by a want of it. Doubtless you will sometimes be tried by the caprices, the irritability, and even the perverseness of such patients, especially if they have been much indulged; and will be obliged, at times, to manifest no little decision. This is, however, not inconsistent with the kindness I have inculcated; and when, at last, the cure is accomplished, you will find yourself fully compensated by a woman's gratitude, and which is never more deep, sincere and lasting, than when it is based on the successful results of professional skill in combination with such conduct as I have suggested.

